# Sunflower *HaGLK* Enhances Photosynthesis, Grain Yields, and Stress Tolerance of Rice

**DOI:** 10.3390/biology14080946

**Published:** 2025-07-27

**Authors:** Jie Luo, Mengyi Zheng, Jiacheng He, Yangyang Lou, Qianwen Ge, Bojun Ma, Xifeng Chen

**Affiliations:** 1 College of Life Sciences, Zhejiang Normal University, Jinhua 321004, China; luojie_1030@163.com (J.L.); 17855881193@163.com (M.Z.); 15770679855@163.com (J.H.); louyangyangzjnu@163.com (Y.L.); mbj@zjnu.cn (B.M.); 2 College of Education, Zhejiang Normal University, Jinhua 321004, China; 3 China-Mozambique Belt and Road Joint Laboratory on Smart Agriculture, Zhejiang Normal University, Jinhua 321004, China

**Keywords:** *HaGLK* gene, photosynthesis, environment stress, crop yield, rice

## Abstract

GOLDEN2-LIKEs (GLKs) are important transcription factors for the chloroplast development influencing photosynthesis, nutrition, senescence, and stress response in plants. Sunflower (*Helianthus annuus*) is a highly photosynthetic plant; here, a *GLK*-homologues gene *HaGLK* was identified from the sunflower genome by bioinformatics. To analyze the bio-function of *HaGLK*, transgenic rice plants overexpressing *HaGLK* (*HaGLK-OE*) were constructed and characterized via phenotype. Compared to the wild-type control rice variety Zhonghua 11 (ZH11), the *HaGLK-OE* lines exhibited increased photosynthetic pigment contents, higher net photosynthetic rates, and enlarged chloroplast area; meanwhile, genes involved in both photosynthesis and chlorophyll biosynthesis were also significantly up-regulated. Significantly, the *HaGLK-OE* plants showed a 12–13% increase in yield per plant. Additionally, the *HaGLK-OE* plants were demonstrated to have improved salt and drought tolerance compared to the control ZH11. Our results indicated that the *HaGLK* gene could play multiple roles in photosynthesis and stress response in rice, underscoring its potential value for improving crop productivity and environmental adaptability in breeding.

## Simple Summary

The rapid growth of the global population and the consequent increase in food demand have made enhancing crop yields a primary focus of agricultural research. Although traditional breeding techniques have improved crop photosynthetic efficiency, their potential appears to be approaching its limit. Consequently, future research should prioritize innovative strategies to further enhance photosynthetic efficiency and stress tolerance, thereby promoting sustainable agriculture. In this study, the *HaGLK* gene was cloned from sunflower and subsequently overexpressed in transgenic rice through genetic transformation. Evaluation of photosynthetic capacity, agronomic traits, and stress tolerance revealed that heterologous expression of *HaGLK* enhances photosynthetic efficiency, crop yields, and stress resilience in rice. These results provide valuable genetic resources and references for breeding new rice varieties with salt tolerance, drought resistance, and high yield and also establish a theoretical foundation for further exploration of the *HaGLK* gene’s function in sunflower.

## 1. Introduction

Photosynthesis is a process in plants using sunlight, carbon dioxide, and water to produce oxygen and glucose. Photosynthesis can directly affect crop yield by converting the glucose into biomass, including grains, fruits, and other harvestable parts. A chloroplast is an organelle in plant cells where photosynthesis occurs, containing chlorophyll and other pigments to transform solar energy into chemical energy. The structure and function of a chloroplast are fundamental to sustaining photosynthesis. Evidence has shown that the accumulation of chlorophyll content in leaves was positively correlated with the plant’s light-absorption capacity, photosynthetic efficiency, and crop yield [1]. The transcription factors GOLDEN2-LIKEs (GLKs) are key regulators for chlorophyll biosynthesis and photosynthesis in plants by regulating *photosynthesis-associated nuclear* genes (*PhANGs*) [2].

GLKs belong to the GARP superfamily, containing a DNA-binding domain (DBD), a proline-rich domain, and a GLK/C-terminal (GCT) box [3]. As of now, it has been discovered that diploid plants typically contain two homologous *GLK* genes [4]. Interestingly, the function of the two GLKs is mostly redundancy in C_3_ plants with only one type of chloroplast in mesophyll cells, such as with Arabidopsis, rice, and tomato [5]. Nevertheless, the chloroplasts in mesophyll cells and bundle-sheath cells are different in C_4_ plants, and the GLKs display unredundancy to determine the differentiation of chloroplasts, such as with maize [6]. Since C_4_ plants exhibit higher photosynthetic efficiency than that of C_3_ plants, ectopic expression of the maize *GLK* genes in rice successfully improved photosynthetic capacity and increased yield of the transgenic plants [7]. In addition, *GLK*s also participate in the stress response of plants. Overexpression of *GLK* genes could enhance plant tolerance to biotic and abiotic stresses, such as drought [8], low temperature [9], or ozone [10]. GLKs can interact with lesion-simulating disease 1 (LSD1) to regulate reactive oxygen species (ROS) production, which leads to programmed cell death (PCD) and immunity to disease [11,12].

Sunflower (*Helianthus annuus*) is a C_3_ plant, which exhibits distinctive photosynthetic efficiency comparable to that of C_4_ plants [13]. Sunflower is also known for its drought and salt tolerance and its ability to grow in barren and arid regions [14,15,16]. Notably, we found that only one *GLK* gene (*HaGLK*) in sunflower is different from the other diploid plants [17]. We hypothesize that overexpression of the *HaGLK* gene in rice may enhance its tolerance to salt stress, drought resistance, and photosynthetic capacity. To test this hypothesis, we constructed the overexpression of *HaGLK* rice transgenic plants and analyzed their photosynthetic performance, agronomic traits, and stress resistance. Here, we show that under the same growth conditions, overexpression of *HaGLK* not only improved the photosynthetic capacity and per-plant yield of rice but also enhanced its tolerance to salt stress and drought resistance.

## 2. Materials and Methods

### 2.1. Plant Materials and Growth Conditions

The variety of sunflower (*Helianthus annuus*) used is ‘Hong yun’. Rice (*Oryza sativa*) *japonica* variety Zhonghua 11 (ZH11) was used as the recipient for genetic transformation of the *HaGLK* gene. For measuring photosynthetic capacity and agronomic traits, rice plants were planted in the field of an experimental farm with a planting density of 20 cm × 20 cm in summer in South China. Water and fertilizer management was conducted according to conventional planting practices. For salt and drought treatment, rice seeds were soaked in water under 37.0 °C for 2 days, and the germinated seeds were transferred into 96-well plates with holes at the bottom and cultured using the Coolaber nutrient solution [18] in a growth chamber under 28 °C (16 h light/8 h dark).

### 2.2. Bioinformatic Analysis

Amino acid sequences of AtGLKs from Arabidopsis (*Arabidopsis thaliana*) were used to search the homology in the genome of sunflower by BLAST in the National Center for Biological Information (NCBI) database (https://www.ncbi.nlm.nih.gov/, accessed on 10 May 2025). Sequence alignments were carried by MEGA5.0 and visualized by GeneDoc2.7, and the conserved motifs of GLK proteins were analyzed using MEME5.4.0 (https://meme-suite.org/meme/ accessed on 10 May 2025).

### 2.3. PCR Cloning of HaGLK

Total RNA of sunflower leaves was extracted using TRIzol reagent (Thermo, San Jose, CA, USA) and reverse transcribed to full-length cDNA by the PrimeScript RT Master Mix reverse transcription kit (TaKaRa, Hangzhou, China). PCR primers were designed according to the coding sequence (CDS) of *HaGLK* (LOC110940494). The sequences of the primer pair are 5′-ATGTTAGCTATTGTGTCACCA-3′ and 5′-TCAAATGCATGTTGGTGGTAT-3′. The KOD FX (TOBOYO, Guangzhou, China) was used for PCR with the 20 µL volume solution the included 0.4 µL each of forward and reverse primer (100 ng μL^−1^), 2 µL cDNA, 1.2 µL DNA polymerase KOD, 2 µL dNTP (2 mM), 2 µL 10× buffer, and 12 µL ddH_2_O. The PCR program was processed as: 94 °C for 2 min; 94 °C for 30 s; 58 °C for 1 min; 72 °C for 30 s, 30 cycles; and 72 °C for 10 min.

### 2.4. Vector Construction and Rice Genetic Transformation

Primer pair, 5′-ggtgttacttaagcttATGTTAGCTATTGTGTCACCAT-3′ and 5′-tgtagtccatggtaccTCAAATGCATGTTGGTGGTAT-3′ (adaptors are underlined), was used to amplify the CDS of *HaGLK* by KOD FX PCR kit (TOBOYO, Guangzhou, China) according to the manufacturer’s instructions. Vector pCAMBLA1300 with a maize (*Zea mays*) ubiquitin (*Ubi*) promoter in the multiple clone site (MCS) was selected. The PCR products and the vector were digested by the restriction enzymes *Hind* III and *Kpn* I (TaKaRa, Hangzhou, China) and recombined by the In-Fusion HD cloning kits (TaKaRa, Hangzhou, China) according to the manufacturer’s instructions. This constructed vector, in which the expression of *HaGLK* was driven by the *Ubi* promoter, was genetically transformed into rice variety ZH11, calli-induced from mature seeds through the method of Agrobacterium-mediated transformation. Callus induction, transformant selection, and seedling regeneration were carried out under continuous light at 32 °C [19]. To identify the positive transgenic plants, the genomic DNA of T_0_ plants was extracted by the CTAB method [20] and tested by PCR mix (CWBIO, Beijing, China) using the primer pair 5′-ATGTTAGCTATTGTGTCACCA-3′ and 5′-TCAAATGCATGTTGGTGGTAT-3′. The thermal cycling protocol comprised an initial denaturation at 95 °C for 60 s, followed by 35 cycles of denaturation at 95 °C for 10 s, annealing at 60 °C for 30 s, and an extension at 72 °C for 30 s, with a final extension step at 72 °C for 5 min.

### 2.5. Gene Expression Analysis

Total RNA of leaves from the positive transgenic plants was extracted using TRIzol reagent (Thermo, San Jose, CA, USA) and reverse transcribed to full-length cDNA by the PrimeScript RT Master Mix reverse transcription kit (TaKaRa, Hangzhou, China) according to the manufacturer’s instructions. Quantitative real-time PCR (qPCR) was performed using the SYBR Green Master Mix kit (TOYOBO, Guangzhou, China). The PCR process was as follows: 95 °C for 60 s, 95 °C for 10 s, 60 °C for 20 s, and 72 °C for 15 s, for a total of 40 cycles. The housekeeping gene *Actin* was used for the standardization control. The primers used for qPCR are listed in Table 1. Each experiment included three biological replicates. The data from the qPCR were analyzed by the 2^−△△Ct^ method [21].

### 2.6. Content Measurement of Photosynthetic Pigments

At the tillering stage, the flag leaves were selected for extraction of photosynthetic pigments. Leaves were cut into 4 mm fragments, weighed at 0.1 g, and incubated with 3 mL 80% acetone (*v*/*v*) in a centrifugal tube under dark conditions for 48 h. Then, the contents of pigments were measured using a spectrophotometer by the method described by Porra et al. [22]. Three biologic replicates were carried out for each sample.

### 2.7. Photosynthetic Assay

At the tillering stage, the net photosynthetic rate (*Pn*) of the flag leaves was measured on sunny day using a LI-COR 6800 photosynthesizer (LI-COR, Lincoln, NE, USA). The light-response curve was measured at CO_2_ concentration of 400 μmol mol^−1^, using the built-in light-response program with varying photon density ranging from 0 to 2800 μmol m^−2^s^−1^.

### 2.8. Ultrastructural Analysis of Chloroplasts

At the heading stage of the rice, the fresh flag leaf samples were collected from the same leaf position for experimental analysis. Subsequently, the protocol described by Feder et al. [23] was employed, utilizing chemical fixation and resin embedding to prepare plant specimens for observation of chloroplast ultrastructure. Three biologic replicates were carried out for each sample. The sizes of chloroplasts were quantified by ImageJ2.1.0.

### 2.9. Statistical Measurement of Rice Agronomic Traits

At the mature stage of the rice, 20 plants from each sample were randomly selected to measure agriculture traits, including plant height, flag-leaf length and width, effective tiller number, panicle length, grains per panicle, seed-setting rate, grain length and width, and thousand-grain weight and yield per plant. The data were statistically analyzed by GraphPad Prism9.5.

### 2.10. Stress Treatments

Germinated rice seeds were germinated with 100 mM NaCl or 15% PEG6000 for 7 days, followed by 3 days recovery in the Coolaber nutrient solution; then, the stem and root lengths were measured. Six biologic replicates were carried out for each sample.

Two-week-old seedlings (n = 48) were transplanted into the 150 mM NaCl or 20% PEG6000 for 7 days in hydroponic systems, followed by 3 days recovery in the Coolaber nutrient solution. Stress tolerance was evaluated by measuring post-recovery survival rates. Survival rate = number of surviving plants/total number of plants × 100%.

## 3. Results

### 3.1. Bioinformatic Identification of HaGLK Gene

**Figure 1:**
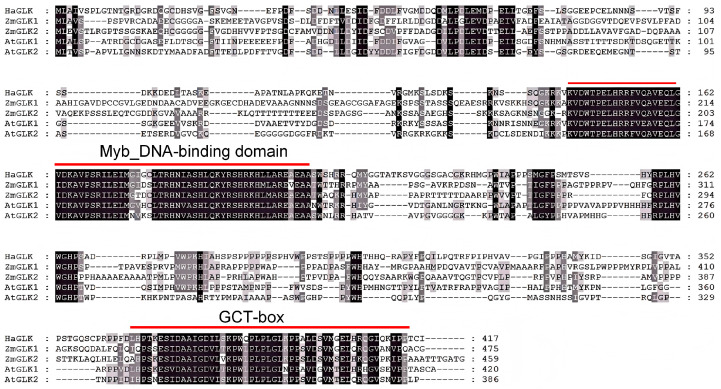
Comparative alignment of HaGLK, ZmGLK, and AtGLK amino acid sequences.

Amino acid (aa) sequences of ZmGLK1 and ZmGLK2 from maize, as well as AtGLK1 and AtGLK2 from Arabidopsis, were respectively used for NCBI BLASTN1.30 in the genome of sunflower, and only one putative *GLK*-like gene (GenBank: LOC110940494) has been found. *HaGLK* is located on chromosome 5 of sunflower, containing six exons. The CDS is 1252 bp in length, encoding a 417 aa protein. By NCBI BLASTP1.30, it showed that the consistencies of protein sequences of HaGLK with ZmGLK1 and ZmGLK2 were 41.79% and 41.28%; HaGLK with AtGLK1 and AtGLK2 were 47.67% and 43.55%, respectively. Sequence alignment with AtGLK and ZmGLK proteins showed that HaGLK has a Myb-type DBD-conserved domain, which recognizes and binds to specific DNA sequences in the promoter region of target genes [24], and a GLK-specific C-terminal GCT box (Figure 1).

### 3.2. Generation and Identification of HaGLK Transgenic Rice

**Figure 2:**
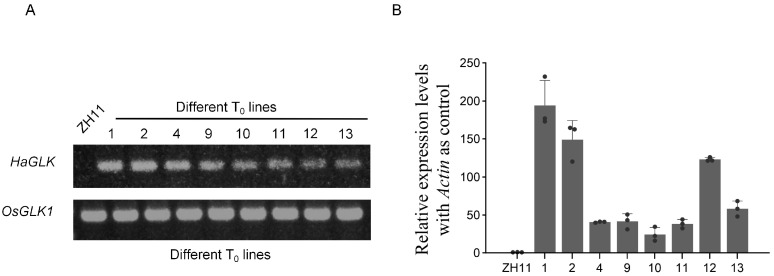
Molecular identification of *HaGLK* transgenic lines. (**A**) PCR identification of *HaGLK* transgenic rice; (**B**) relative expression of *HaGLK* by quantitative real-time PCR (qPCR). Each dot represents a biological replicate.

The full-length cDNA of the *HaGLK* gene was amplified by PCR and transformed into rice variety ZH11, in which *HaGLK* was driven by the *Ubi* promoter. From twenty-one T_0_ plants, eight positive ones were firstly identified by PCR using the primers of a *hygromycin*-resistant gene. Then, the eight transgenic plants were further confirmed by the PCR using *HaGLK* and *OsGLK1* gene-specific primers. Results show that the *OsGLK* gene can be amplified from ZH11 and the transgenic plants, while the *HaGLK* gene can only be amplified from the transgenic plants (Figure 2A and Figure S1). Subsequently, the *HaGLK* gene transcription levels were tested by qPCR. Results show that the expression of *HaGLK* vary in different transgenic lines, and two lines, *HaGLK-OE1* and *HaGLK-OE2*, exhibited higher expression levels than those of other lines (Figure 2B), which were selected for further characterization.

### 3.3. Photosynthetic Capacity Analysis in HaGLK Transgenic Rice

**Figure 3:**
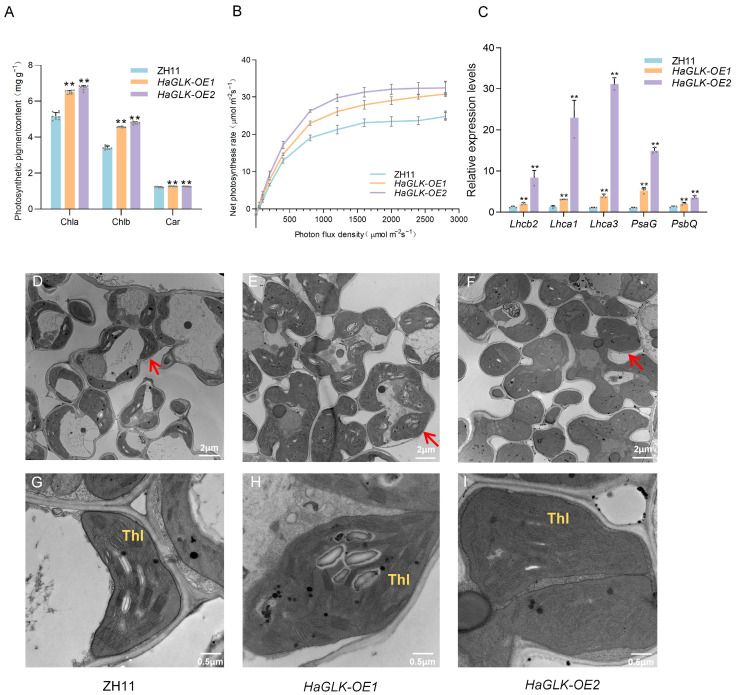
Photosynthesis-related indicator analysis of ZH11 and *HaGLK* transgenic lines. (**A**) Photosynthetic pigment content of flag leaves at the tillering stage (n = 9 biological replicates); (**B**) net photosynthesis rate curves fitted by the built-in light-response program (LI-COR 6800) with the CO_2_ concentration of 400 μmol mol^−1^ (n = 3 biological replicates); (**C**) relative expression of photosynthesis-related genes by qPCR (n = 3 biological replicates); (**D**–**I**) transmission electron micrographs of flag-leaf cross-sections of ZH11 and *HaGLK* transgenic lines; (**D**–**F**) chloroplasts in mesophyll cells, with arrows pointing to representative chloroplasts (bar = 2 μm); (**G**–**I**) enlarged micrographs of chloroplasts in mesophyll cells (bar = 0.5 μm). Thl: a stack of granal thylakoids. Data are means ± SD (Student’s *t*-tests, ** *p* < 0.01). Each dot represents a biological replicate.

At the tillering stage, photosynthetic pigment contents in leaves of the *HaGLK-OE1* and *HaGLK-OE2* transgenic lines were significantly higher than in ZH11 (Figure 3A). The expression levels of photosynthesis and chlorophyll biosynthesis-related genes in *HaGLK-OE1* and *HaGLK-OE2* transgenic lines were significantly higher than in ZH11 (Figure 3B), as shown by qPCR. Transmission electron microscopy (TEM) analysis revealed that the chloroplast area of transgenic lines *HaGLK-OE1* and *HaGLK-OE2* increased by 30% and 54.3%, respectively, while the number of thylakoids per chloroplast rose by 38.8% and 36.7%, respectively (Figure 3D–I). At the heading stage, the net photosynthetic ratio (*Pn*) of flag leaves from the two transgenic lines and ZH11 was measured, and the *Pn* of transgenic lines was significantly higher than that of ZH11 with the increasing light intensity (Figure 3C).

### 3.4. Agronomic Trait Survey of HaGLK Transgenic Rice

**Figure 4:**
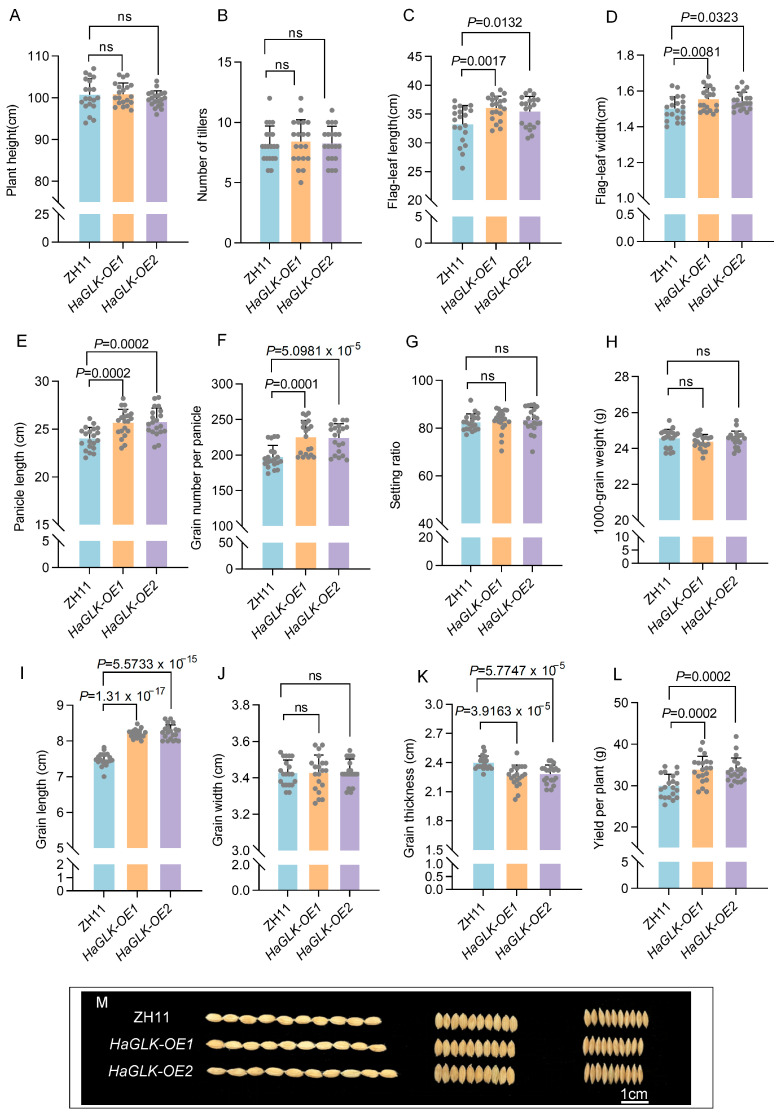
Agronomic trait analysis of ZH11 and *HaGLK* transgenic lines. (**A**) Plant height; (**B**) number of tillers; (**C**) flag-leaf length; (**D**) flag-leaf width; (**E**) panicle length; (**F**) grain number per panicle; (**G**) setting ratio; (**H**) 1000-grain weight; (**I**) grain length; (**J**) grain width; (**K**) grain thickness; (**L**) yield per plant; (**I**–**K**) ten grains in length (**I**), width (**J**), and thickness (**K**) between ZH11 and *HaGLK* transgenic lines; (**M**) comparison of grain shape (bar = 1 cm). Data are means ± SD (Student’s *t*-tests, ns: non-significance, n = 20 biological replicates). Each dot represents a biological replicate.

The agronomic traits of *HaGLK-OE1* and *HaGLK-OE2* transgenic lines were statistically evaluated compared to ZH11. The results show that there were no significant changes in plant height and tiller number (Figure 4A,B). However, the flag-leaf length and flag-leaf width increased in the transgenic lines (Figure 4C,D), which effectively expands the area of photosynthesis of plants. Notably, the panicle length and grain number per panicle of the transgenic lines significantly increased (Figure 4E,F), while the seed-setting ratio and 1000-grain weights of the transgenic lines demonstrated no significant change (Figure 4G,H), which results in enhancement of yields per plant by 13.1% and 12.6% for *HaGLK-OE1* and *HaGLK-OE2*, respectively (Figure 4L). Interestingly, the grain shape of *HaGLK-OE* transgenic lines has become longer and thinner, because their grain length increased and their grain thickness decreased with no change of grain width (Figure 4I–K).

### 3.5. Stress Tolerance Analysis of HaGLK Transgenic Rice

Abiotic stresses such as salt and drought have been shown to severely inhibit the normal growth and development process of rice, which is an important factor limiting rice yield [25]. To further investigate the stress tolerance of *HaGLK* transgenic rice, stress treatments were performed on the transgenic rice lines and ZH11. Under normal conditions, the growth status of *HaGLK-OE1* and *HaGLK-OE2* transgenic seedlings showed no phenotypic differences compared to ZH11, indicating that heterologous expression of the *HaGLK* gene did not affect the growth and development of rice seedlings during the early stage.

**Figure 5:**
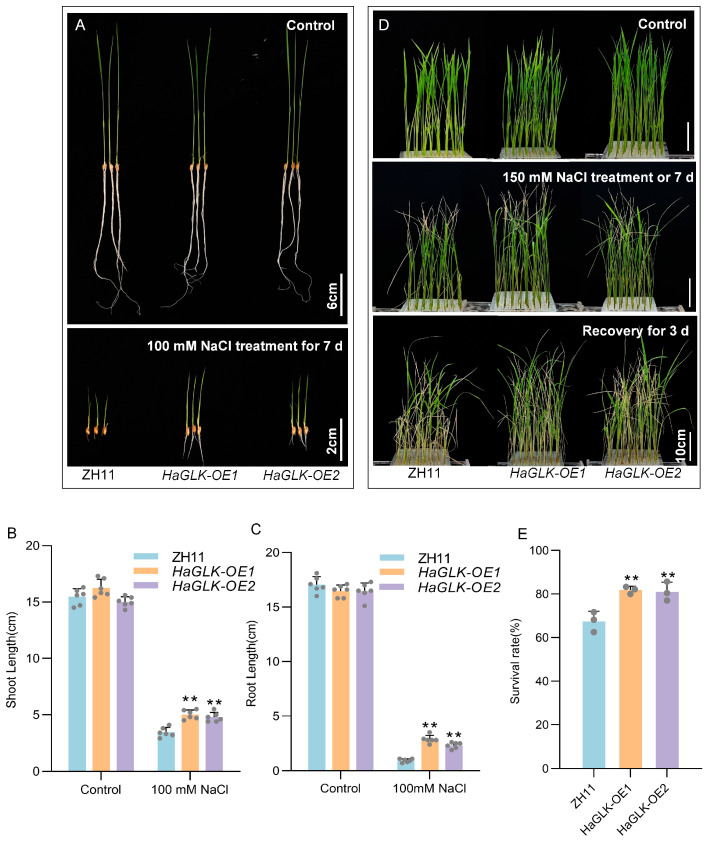
Phenotypic analysis of the *HaGLK* transgenic lines under NaCl treatment. (**A**) Germination stage—sprouting seeds were supplemented with 100 mM NaCl (bar = 2 cm) and control without any additional additives (bar = 6 cm); (**B**,**C**) shoot and root length of plants under 100 mM NaCl treatment (n = 6 biological replicates); (**D**) seedling stage—2-week-old seedlings were supplemented with 150 mM NaCl and control without any additional additives (bar = 10 cm); (**E**) survival rates of seedlings with 150 mM NaCl (n = 3 biological replicates). Data are means ± SD (Student’s *t* tests, ** *p* < 0.01). Each dot represents a biological replicate.

When sprouting seeds at the germination stage were treated with a 100 mM NaCl solution for 7 days, measurements showed that the shoot and root lengths of *HaGLK-OE1* and *HaGLK-OE2* transgenic lines were found to be significantly higher than those of ZH11 (Figure 5A–C). Subsequently, 2-week-old seedlings were subjected to 150 mM NaCl solution for 7 days, followed by 3 days of recovery. ZH11 seedlings exhibited severe symptoms such as leaf desiccation, bending, wilting, and even plant death, whereas *HaGLK-OE1* and *HaGLK-OE2* transgenic lines showed much milder symptoms of leaf desiccation, bending, and wilting (Figure 5D). Statistical analysis of survival rates revealed that the survival rates of *HaGLK-OE1* and *HaGLK-OE2* transgenic lines were 82% and 80%, respectively, significantly higher than the 64% survival rate of ZH11 (Figure 5E).

**Figure 6:**
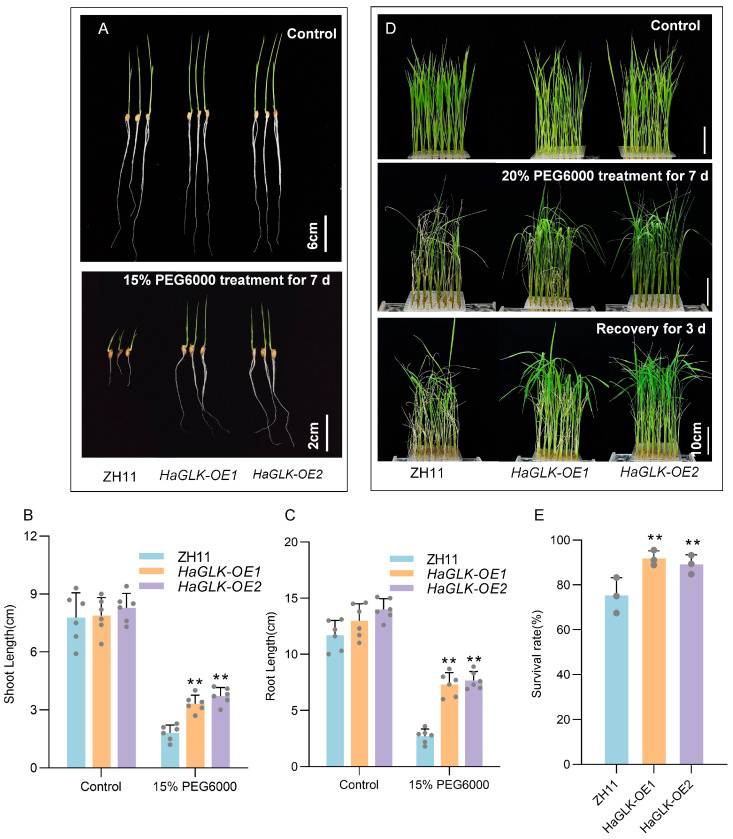
Phenotypic analysis of the *HaGLK* transgenic lines under PEG6000 treatment. (**A**) Germination stage—sprouting seeds were supplemented with 15% PEG6000 (bar = 2 cm) and control without any additional additives (bar = 6 cm); (**B**,**C**) shoot and root length of plants under 15% PEG6000 treatment (n = 6 biological replicates); (**D**) seedling stage—2-week-old seedlings were supplemented with 20% PEG6000 and control without any additional additives (bar = 10 cm); (**E**) survival rates of seedlings with 20% PEG6000 (n = 3 biological replicates). Data are means ± SD (Student’s t tests, ** *p* < 0.01). Each dot represents a biological replicate.

Under drought stress, *HaGLK-OE1* and *HaGLK-OE2* transgenic lines displayed strong tolerance. At the seed germination stage, after 7 days of treatment with 15% PEG6000, the growth of the ZH11 was significantly inhibited, in which the shoot and root lengths were shorter and had smaller leaves compared to the transgenic lines (Figure 6A–C). When 2-week-old seedlings were subjected to 20% PEG6000 drought stress for 7 days, the *HaGLK-OE1* and *HaGLK-OE2* transgenic lines showed good tolerance, as most leaves remained normal growth except for slight necrosis and drooping at the leaf tips; on the contrary, the ZH11 seedlings showed significant curling, wilting, and lodging after stress treatment. After 3 days of recovery, most seedlings of *HaGLK-OE1* and *HaGLK-OE2* transgenic lines resumed normal growth (Figure 6D). The survival rates of *HaGLK-OE1* and *HaGLK-OE2* transgenic lines were 92% and 89%, respectively, significantly higher than the 75% survival rate of ZH11 (Figure 6E). These results indicate that heterologous expression of *HaGLK* enhances the salt and drought tolerance of rice plants.

## 4. Discussion

In the face of the dual pressures of global population growth and limited arable land resources, it is imperative to stabilize the yield of rice as one of the major food crops. Research demonstrates that GLKs transcription factors regulate chloroplast biogenesis and function by activating chlorophyll biosynthesis and photosynthesis-related genes [26]. Constitutive *GLK* expression enhances chloroplast development in photosynthetic tissues [27] and promotes photosynthetic activity in non-green cells [28]. Recent studies reveal that the PsbS subunit protein of photosystem II overexpression in rice improves non-photochemical quenching under fluctuating light, boosting photosynthetic efficiency and yield [29]. Specifically, OsGLK1 and OsGLK2 bind to promoters of light-harvesting *Lhca*/*Lhcb* genes to transactivate expression [30], while the transcription factor OsNFYB7 from the nuclear factor Y family inhibits GLK1-mediated transcriptional activation, diminishing chlorophyll buildup in rice embryos [31]. Consistent with these mechanisms, our findings show that the overexpressed *HaGLK* gene in rice up-regulates the expression of light-harvesting genes and photosystem subunits, significantly increasing photosynthetic pigments, net photosynthetic rate, and chloroplast size in leaves (Figure 3). However, *HaGLK* overexpression caused neither developmental delays nor enhanced photosynthesis in non-green tissues during whole-plant growth.

Heightened chlorophyll accumulation in foliage is linked to augmented photosynthetic performance and greater agricultural yield [1]. GLKs exhibit broad application prospects in crop breeding, where regulating the expression of photosynthesis-associated GLKs represents an effective strategy for enhancing agricultural productivity. In Arabidopsis, it was observed that high expression of *AtGLK1* through leaf- and rachis wall-specific promoters resulted in significant enhancements in photosynthesis and an increase in seed oil content by 2.88% and 10.75%, respectively [32]. In oil-seed rape (*Brassica napus*), overexpression of *BnGLK1a* resulted in a 10% increase in the 1000-grain weight of seeds [33]. Furthermore, overexpression of *ZmGLK1* and *ZmGLK2* in rice led to a 30% to 40% increase in yield, while expression driven by the native promoter can enhance yields by 47% to 70% [34,35]. In this study, we found that heterologous expression of *HaGLK* in rice and the yield per plant of *HaGLK-OE1* and *HaGLK-OE2* increased by 13.06% and 12.60%, respectively (Figure 4L). Agronomic trait investigations revealed that the leaf length and width of *HaGLK* transgenic rice increased significantly. Although the *HaGLK* gene altered the grain shape of rice, there was no significant difference in the seed-setting rate compared with ZH11 (Figure 4). This indicates that *HaGLK* transgenic rice increased the total yield by increasing the number of grains per panicle. Meanwhile, *HaGLK* also affected the increase in panicle length, proving that *HaGLK* plays an important role in the growth and development of rice. These research results provide a scientific basis for further studies on the functions of GLK and its potential applications in rice breeding.

The growth status of leaves is an important indicator of plants’ response to abiotic stresses [36]. When plants exhibit greater resistance to drought and salt, the chlorophyll content stays higher, and with a stronger membrane stability, it can make better use of light energy [37]. Under salt and drought treatment, leaves are prone to water loss, causing a reduction in turgor pressure and leading to wilting or death. GLKs play a significant role in plant responses to abiotic stress, and genome-wide analyses predict a conserved function of GLKs in the abiotic stress responses of various crops [34,38]. Under drought- and cold-stress conditions, the growth and yield of the cotton (*Gossypium hirsutum*) *ghglk1* mutant are significantly affected, and heterologous expression of *GhGLK1* in Arabidopsis can enhance tolerance to drought and cold stress [9]. The chimeric repressor *GLKs*-SRDX down-regulates K^+^ channel genes in Arabidopsis to enhance ozone tolerance [10]. The *AhGLK1* of peanut (*Arachis hypogaea*) was transformed into the *glk1glk2* mutant of Arabidopsis and affected the morphological development and photosynthesis of Arabidopsis, rescuing the pale green phenotype of the mutant. The survival rate of the mutant during the drought recovery process has been improved [39]. Additionally, GLKs are involved in regulating plant sensitivity to ABA and ion-channel activity. They enhance seedling sensitivity to osmotic stress mediated by the ABA signaling pathway by regulating WRKY40 [40]. Under NaCl and PEG6000 treatment, *HaGLK* transgenic rice exhibited significantly longer roots and stems during the germination phase, the degree of leaf wilting was significantly lower compared to ZH11, and the survival rate of seedlings was also significantly improved (Figure 5 and Figure 6). It indicates that under stress conditions, *HaGLK* transgenic rice rapidly adjusts stomatal behavior by closing stomata to minimize water dissipation, thereby upholding cellular turgor pressure, retaining leaf erectness, supporting routine metabolism, and improving resistance to salinity and drought stresses. Sunflowers, as C_3_ plants, exhibit photosynthetic efficiency comparable to that of C_4_ plants due to their ability to sustain photosynthesis with lower CO_2_ concentrations [13]. Thus, even with closed stomata and decreased CO_2_ absorption, chlorophyll levels in *HaGLK* transgenic rice increase, allowing photosynthetic efficiency to be preserved. Further exploration of the mechanisms by which heterologous expression of *HaGLK* affects chloroplast development in rice and the responses to salt and drought stress is important for the stable increase of rice yield and enhancement of its stress resistance.

## 5. Conclusions

In this study, transgenic rice lines overexpressing *HaGLK* were generated via genetic transformation. Comprehensive analyses of photosynthetic capacity, agronomic traits, and stress tolerance demonstrated that *HaGLK*-overexpressed transgenic rice significantly increased photosynthetic pigment content, net photosynthetic rate, chloroplast size, and other photosynthesis-related parameters as well as per-plant yield, while markedly enhancing tolerance to salt stress and drought resistance. Future research should aim to elucidate the mechanisms by which heterologous *HaGLK* expression modulates photosynthetic efficiency and stress responses in rice. These findings will provide vital genetic resources for breeding salt- and drought-tolerant rice cultivars, while establishing a theoretical foundation for functional studies of the *HaGLK* gene in sunflower.

## Figures and Tables

**Table 1:** Sequences of primer used for qPCR.

Gene	Forward Primer (5′→3′)	Reverse Primer (5′→3′)
*HaGLK*	CACTCACCATCACCACCACCA	GGATACCAGGGACCGCTACAT
*Lhca1*	GAGTTCGTCGCCATCGCCTTCG	CGCCCGTTCTTGATCTCCTTGAGC
*Lhca3*	GTTCGCATCCAAGCAGTCCCT	CCGTTGAACACCTCGCCGTAG
*Lhcb2*	TGTTCTCCATGTTCGGCTTCTTCG	GCGTATGCCCAGGCGTTGTT
*PsaG*	AGCGGGAGAACGTGGCGAAGCA	GCAGGTGGCGAGGATGTAGTAGGC
*PsbQ*	GAATTGAAGGGCTGCATTTGT	CCGAAGATCCCGAAGAAGGT
*Actin*	TGGCATCTCTCAGCACATTCC	TGCACAATGGATGGGTCAGA

## Data Availability

The data presented in this publication are available on request from the corresponding author.

## Supplementary Materials

The following supporting information can be downloaded at: https://www.mdpi.com/article/10.3390/biology14080946/s1. Figure S1: Electrophoretic image of *HaGLK* transgenic rice by PCR identification.
