# Supplementary figures and images for: Sunflower HaGLK Enhances Photosynthesis, Grain Yields, and Stress Tolerance of Rice
Source: Biology (Basel). 2025 Jul 27;14(8):946. doi: 10.3390/biology14080946 (PMC12384029; doi:10.3390/biology14080946)

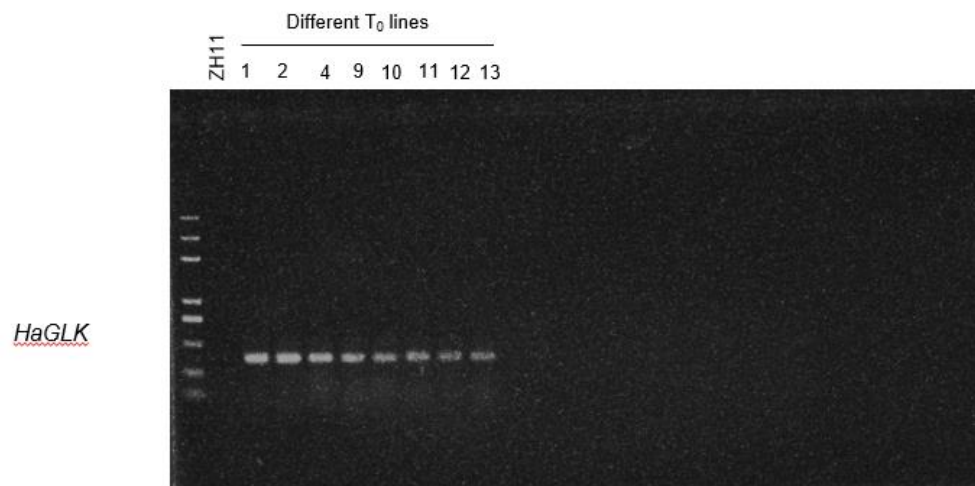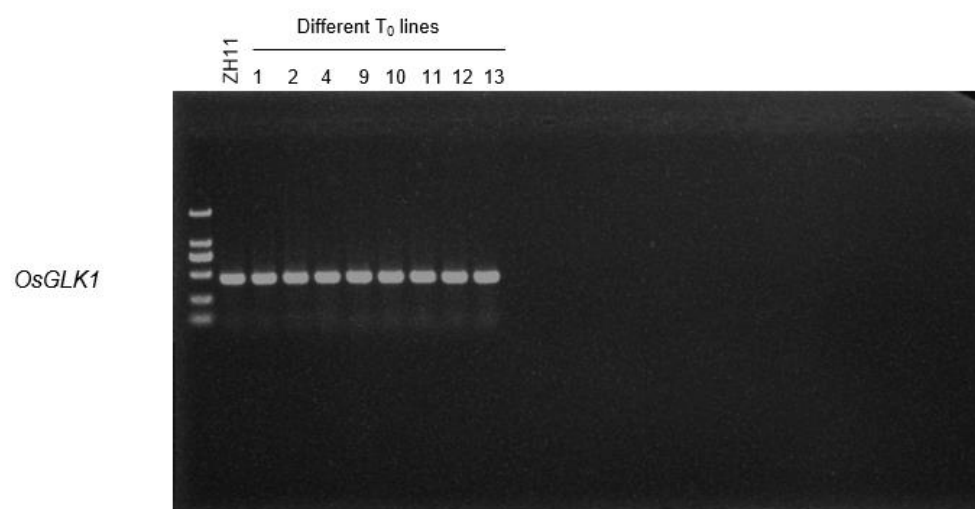

PCR identification of *HaGLK* transgenic rice

Supplement: Supplementary file 1 [file biology-14-00946-s001.zip › biology-3732344-supplementary.pdf]
